# The Antarctic sea ice alga *Chlamydomonas* sp. ICE-L provides insights into adaptive patterns of chloroplast evolution

**DOI:** 10.1186/s12870-018-1273-x

**Published:** 2018-04-03

**Authors:** Zhenhua Zhang, Meiling An, Jinlai Miao, Zhiqiang Gu, Chang Liu, Bojian Zhong

**Affiliations:** 1 Jiangsu Key Laboratory for Biodiversity and Biotechnology, College of Life Sciences, Nanjing Normal University, Nanjing, China; 2 Medical College, Qingdao University, Qingdao, China; 3 Key Laboratory of Marine Bioactive Substance, The First Institute of Oceanography, State Oceanic Administration, Qingdao, China; 4 State Key Laboratory of Natural Medicines, School of Life Science and Technology, China Pharmaceutical University, Nanjing, China

**Keywords:** Antarctic ice algae, Positive selection, Photosynthesis, Convergent evolution

## Abstract

**Background:**

The ice alga *Chlamydomonas* sp. ICE-L is the main contributor to primary productivity in Antarctic sea ice ecosystems and is well adapted to the extremely harsh environment. However, the adaptive mechanism of *Chlamydomonas* sp. ICE-L to sea-ice environment remains unclear. To study the adaptive strategies in *Chlamydomonas* sp. ICE-L, we investigated the molecular evolution of chloroplast photosynthetic genes that are essential for the accumulation of carbohydrate and energy living in Antarctic sea ice.

**Results:**

The 60 chloroplast protein-coding genes of *Chlamydomonas* sp. ICE-L were obtained, and the branch-site test detected significant signatures of positive selection on *atpB*, *psaB*, and *rbcL* genes in *Chlamydomonas* sp. ICE-L associated with the photosynthetic machinery. These positively selected genes were further identified as being under convergent evolution between *Chlamydomonas* sp. ICE-L and the halotolerant alga *Dunaliella salina*.

**Conclusions:**

Our study provides evidence that the phototrophic component of *Chlamydomonas* sp. ICE-L exhibits adaptive evolution under extreme environment. The positive Darwinian selection operates on the chloroplast protein-coding genes of Antarctic ice algae adapted to extreme environment following functional-specific and lineages-specific patterns. In addition, three positively selected genes with convergent substitutions in *Chlamydomonas* sp. ICE-L were identified, and the adaptive modifications in these genes were in functionally important regions of the proteins. Our study provides a foundation for future experiments on the biochemical and physiological impacts of photosynthetic genes in green algae.

**Electronic supplementary material:**

The online version of this article (10.1186/s12870-018-1273-x) contains supplementary material, which is available to authorized users.

## Background

The Antarctic area is a particularly inclement environment with extremely low temperatures. Seasonal fluctuations in temperatures from winter to summer (from − 80 °C to 5 °C), high UV-B radiation and alternating long periods of sunlight and darkness are major challenges for organisms to survive in Antarctica [1]. The Antarctic sea ice is an important constituent of the polar extreme environments, and it comprises a system of concentrated brine channels under low temperature, high salinity and low light conditions [2]. Survival in this harsh environment demands complex adaptations in physiology and metabolism. Antarctic ice algae thrive in the sea ice, and their prolific growth makes them the main contributors to primary productivity in sea ice ecosystems [2, 3]. It has been reported that Antarctic ice algae form a series of physiological and biochemical mechanisms to adapt to the extreme environments [1, 4–7]. The rapid growth of molecular data provides us with a new approach to study adaptive mechanisms. Uncovering adaptive strategies at the molecular level can reveal evolutionary patterns and provide novel insights into adaptability of algae to extreme environments.

Green algae can convert inorganic carbon into organic compounds by photosynthesis in chloroplasts, and photosynthetic systems are sensitive to abiotic environmental stresses (low light, low temperature and high salinity). The chloroplast is the photosynthetic reaction center, and the chloroplast genomes are useful resources for evolutionary studies on photosynthesis [8, 9]. This maternally inherited, non-recombining chloroplast genome encodes many important subunits of photosynthetic systems (some subunits are encoded by the nuclear genes), and the chloroplast genome sizes vary among different green algae [10, 11]. Comparative genomic analyses of three model organisms (*Arabidopsis thaliana*, *Chlamydomonas reinhardtii* and *Thermosynechococcus elongatus*) indicate that 48 chloroplast genes fall into two broad groups: 29 genes for the photosynthetic apparatus; 19 genes for components of the chloroplast genetic system. These 29 chloroplast genes encode five photosynthetic complexes: photosystem I (PSI), photosystem II (PSII), Cytochrome *b*_6_*f* (Cyt*b*_6_*f*), ATP synthesis and Rubisco. The protein complexes PSI, PSII and Cytochrome *b*_6_*f* (Cyt*b*_6_*f*) are membrane-intrinsic. These complexes form the photosynthetic electron transport chain which can transfer electron from PSII to oxidized PSI reaction centers where NADP^+^ is reduced to NADPH. Both PSI and PSII are stress sensitive, and genes encoding the two complexes can respond to abiotic stress at transcriptional and post-transcriptional levels [12, 13]. During electron transfer, proton gradients are built and utilized by chloroplastic ATP synthase. The ATP is generated that can be used in the process of photosynthetic fixation of CO_2_. Rubisco is extrinsic to the membrane and catalyzes the first step of the reductive pentose phosphate pathway of carbon dioxide assimilation. High concentration of NaCl and low temperature may limit the activity of Rubisco, which would inhibit the fixation of CO_2_ [14, 15]. The *rbcL* gene encoding Rubisco in many plants adapted to extreme environments shows various amino acid substitutions in Rubisco that influence abiotic stress responses [14].

Antarctic plants have evolved a number of adaptive molecular mechanisms in five complexes of the photosynthetic system to survive in the harsh environment [16–18]. It is reported that *Colobanthus quitensis* (angiosperm) living in Antarctica has adapted to maintain faster repair rate of the photosynthetic system [19]. The Antarctic macroalgae (e.g. *Himantothallus grandifolius* and *Desmarestia anceps*) evolved high photosynthetic efficiency, low light requirements for photosynthesis and notable UV tolerance [20]. Interestingly, the Antarctic ice diatom *Amphiprora kufferathii* utilizes epiphytic bacteria to consume the reactive oxygen species (ROS) produced during photosynthesis [21]. The increasing molecular data of Antarctic plants will help to investigate the adaptation of photosynthesis to the extreme environment of Antarctica from an evolutionary perspective. It has been reported that positively selected and rapidly evolving genes in animals contributed to extreme environment adaptations [22–24]. In plants, the evidence of positive selection in the Rubisco gene (involved in carbon fixation) from mosses has been associated with its adaptation to the declining levels of atmospheric CO_2_ since their origination in the Ordovician [25]. The genes related to the photosynthetic machinery in an endolithic green alga *Ostreobium quekettii* show strong purifying selection due to its low light lifestyle [26]. The genomic analyses of salt-tolerant *Populus euphratica* demonstrate rapid evolution in genes encoding photosynthetic electron transport chain [27].

The green alga *Chlamydomonas* sp. ICE-L is isolated from the floating sea ice with an optimum growth temperature range of 4–10 °C. As the primary productivity of the Antarctic sea ice, *Chlamydomonas* sp. ICE-L evolved specific morphological characteristics as adaptation to the extreme environment, such as the changes in pigments, lipids and fatty acids content for maintaining the stability of the thylakoid membranes and the normal physiological function of the chloroplast [7]. The particularly harsh environment in Antarctica may leave footprints in the photosynthetic genes of *Chlamydomonas* sp. ICE-L by changing rates of molecular evolution. However, there is little genetic evidence on the adaptation to the extreme environment regarding photosynthesis of this alga.

There are other green algae that potentially adapt to similar environmental stress. *Coccomyxa subellipsoidea* C-169 is a small elongated non-motile unicellular green alga isolated in the polar summer of 1959/60 at Marble Point, where there were large moist areas covered with a thick mat of the thalloid *Nostoc commune* [28], and the major environmental factors of *Coccomyxa subellipsoidea* C-169 are low light and low temperature. *Chlorella* sp. ArM0029B is isolated from drift ice in the Arctic region [29], and low temperature is regarded as the main environmental stress. *Dunaliella salina* is a halotolerant alga thriving in extreme saline environments [30], and high salinity is the abiotic stress for *Dunaliella salina*. These green algae as well as *Chlamydomonas* sp. ICE-L living in extreme environments are ideal organisms to comparatively investigate adaptive strategies in different abiotic stresses.

In this study, we sequenced 60 chloroplast protein-coding genes of *Chlamydomonas* sp. ICE-L, and used other available chloroplast genomes of aforementioned green algae living in similar extreme environments (low light, low temperature and high salinity) to investigate the potential genetic basis of adaptation to abiotic stress in the photosynthetic machinery of *Chlamydomonas* sp. ICE-L. Adaptive evolution appears to target the protein-coding components of the chloroplast in a function (photosynthetic components) and lineage specific manner, and the adaptive modifications in these positively selected genes were in functionally important regions. Our analyses revealed signatures of positive selection and convergent evolution among the chloroplast protein-coding genes of Antarctic ice algae, supporting the notion that the adaptation to extreme environments in algae are associated with the altered patterns of selection on chloroplast proteins.

## Methods

### Algal samples and DNA sequencing

The strain of *Chlamydomonas* sp. ICE-L was obtained from the floating sea ice near the Zhongshan Station of Antarctica (China) during the Chinese 18th Antarctic Science Exploration from 2001 to 2002. The *Chlamydomonas* sp. ICE-L was isolated from Antarctic sea ice and monoclonal cultured in the lab. Cells were grown in Provasoli seawater medium [31] under a photon flux density of 40 μmol photons m^− 2^ s^− 1^, 14 L: 10D cycle, and temperature of 8 °C. Total genomic DNA was extracted using the DNeasy Plant Mini Kit (QIAGEN Bio-Tec), and sequenced using Illumina HiSeq™ 2000 platform.

### Chloroplast genes assembly and annotation

A total of 6130 Mb clean reads (125-bp paired-end) were generated by removing adapters, poly-N and low quality reads using in-house perl scripts. All downstream analyses were based on the clean reads. We performed de novo assembly using SPAdes software [32] with the parameter “--plasmid”, “--careful” and other default settings. We created a local database of published chloroplast genomes of green algae, and performed similarity searches to identify the scaffolds from the chloroplast genome using BLAST with a cut-off E-value of 1 × 10^− 10^ [33]. We further used Geneious (version 9.0.4, http://www.geneious.com/) to annotate the chloroplast genes, and confirmed the gene annotations using the DOGMA [34] online tools (http://dogma.ccbb.utexas.edu/).

### DNA alignment and phylogenetic inference

We generated multiple sequence alignments using two different programs: MUSCLE [35] and PRANK [36]. We aligned 60 chloroplast protein-coding genes using six Chlorophyceae and seven Trebouxiophyceae green algae (Table 1). Nucleotide sequences were aligned at the codon level with the option “-codon” using MUSCLE and at the protein level with the option “-translate” using PRANK. Stop codons were removed from the sequences prior to alignment. Each aligned nucleotide sequence was trimmed to exclude poorly aligned positions using Gblocks0.91b with default parameter [37], and concatenated into a single data set using Geneious. Two alignments were almost identical except for a few sites. We used the aligned data produced by PRANK in the subsequent analyses as PRANK has lower false-positive rates [38]. The *rpoB1*, *rpoB2* and *rpoC* genes were excluded due to alignment ambiguities according to visual assessment.

**Table 1:** The 13 green algae used in this study

Order	Taxa	Accession number
Chlorophyceae	*Dunaliella salina*	[GenBank: NC_016732]
	*Chlamydomonas applanata*	[GenBank: KT625417]
	*Microglena monadina*	[GenBank: KT624717 - KT624805]
	*Chlamydomonas* sp. ICE-L	[GenBank: MF280291 - MF280350]
	*Chlamydomonas reinhardtii*	[GenBank: NC_005353]
	*Volvoxcarteri* f. nagariensis	[GenBank: GU084820]
Trebouxiophyceae	*Paradoxia multiseta*	[GenBank: KM462879]
	*Choricystis parasitica*	[GenBank: KM462878]
	*Coccomyxa subellipsoidea*	[GenBank: NC_015084
	*Dicloster acuatus*	[GenBank: NC_025546]
	*Parachlorella kessleri*	[GenBank: NC_012978]
	*Chlorella* sp. ArM0029B	[GenBank: KF554427]
	*Chlorella vulgaris*	[GenBank: NC_001865]

The phylogenetic trees were reconstructed based on the nucleotide data with GTRGAMMA model and amino acid data with PROTGAMMAAUTO model using RAxML v8.2 [39] with a rapid bootstrap search of 1000 replicates. The 3rd codon positions are problematic to phylogenetic inference because of high saturation [40], then only 1st and 2nd codon positions of nucleotide data are used for phylogenetic analyses to minimize negative effects of saturation [41].

### Identification of genes under positive selection

Branch-specific estimates of rates of synonymous (dS) and non-synonymous substitutions (dN) were calculated under the basic model (model = 0, Nsites = 0, which assumes no site-wise or branch-wise dN/dS variation). We discarded 11 genes with high dS value to avoid misestimation of dN/dS. The dS values of remaining 46 genes were below 1 for nearly all branches, ensuring sufficient phylogenetic resolution to accurately estimate dN/dS across the phylogeny [42] (Additional file 1: Table S1). Alignment gaps and uncertainties were deleted to avoid false positives [43]. The codon frequencies were determined by the F3 × 4 model. The remaining 46 genes were divided into two functional classifications (photosynthesis and genetic system), and were separately concatenated. Three different codon-based likelihood models (branch-specific, site-specific and branch-site models) were used to explore the selection patterns and identify positive selection on each of the 46 protein-coding genes and two concatenated data. The branch and branch-site model tests need to assign foreground branch based on a priori knowledge, and incorrect assignments may compromise the power of the test. The goal of our study is to explore the role of positive selection in the adaptive patterns of Antarctic sea ice algae, thus four selected algae adapted to extreme environments are used to perform the selection analyses.

To detect adaptive evolving genes in *Chlamydomonas* sp. ICE-L, we first conducted the branch model analyses using Codeml in PAML. The non-synonymous to synonymous rate ratio ω (dN/dS) among branches indicates changes in patterns of natural selection, where ω = 1, ω < 1 and ω > 1 correspond to neutral evolution, purifying and positive selection, respectively [44]. We used three starting ω values (0.5, 1 and 2) to avoid potential local optima. The null model specified one ω for the entire phylogenetic tree, whereas the alternative model allowed two different ω values for *Chlamydomonas* sp. ICE-L and background lineages. Furthermore, the other three green algae (*Coccomyxa subellipsoidea* C-169, *Chlorella* sp. ArM0029B and *Dunaliella salina*) with extreme living conditions were specified as foreground and repeated branch-model test. The likelihood ratio test (LRT) with a χ^2^ distribution was used to determine which models were statistically different from the null model at a threshold of *P* < 0.05. We applied the FDR correction to the *P* values for the multiple tests performed with a significance level of 0.05 [45].

The site-specific model assumes that selection pattern varies among sites in the alignment but not among branches in the phylogeny. We used a pair of site model comparisons to test for positive selection. The alternative models allow for site-specific positive selection, but the null models do not (M8 vs. null M8a) [44]. LRT statistics were compared to a χ^2^ null distribution with the corresponding degrees of freedom, and the FDR correction was applied to the *P* values calculated above to account for the multiple tests performed with a significance level of 0.05 [45].

To detect evidence of positive selection on specific sites along specific lineage, the improved branch-site model (model = 2, Nsites = 2, fixed omega = 0, omega = 2) implemented in the Codeml program [44] were compared with the null model (model = 2, Nsites = 2, fixed omega = 1, omega = 1). The alternative model assumes four categories of sites: the first two assume sites under purifying selection (0 ≤ ω_0_ ≤ 1) or neutrality (ω_1_ = 1) across the phylogeny, whereas the remaining two assume sites under either purifying selection or neutrality along the background but with positive selection (ω_2_ > 1) in foreground. The null model fixed the ω_2_ = 1 in foreground. Because branch-site models do not allow for multiple foreground branches, the branch leading to *Chlamydomonas* sp. ICE-L, *Coccomyxa subellipsoidea* C-169, *Chlorella* sp. ArM0029B and *Dunaliella salina* was separately chosen as foreground branch in each analysis. The likelihood ratio test (LRT) with a χ^2^ distribution was used to determine which models were statistically different from the null model at a threshold of *P* < 0.05. We applied the FDR correction to the *P* values calculated above to account for the multiple tests performed with a significance level of 0.05 [45]. Bayes empirical Bayes (BEB) method was used to statistically identify sites under positive selection with posterior probabilities ≥0.95 [46].

### Convergent and parallel evolution analyses

To determine whether similar patterns of adaptive evolution occurred in distant lineages habitually exposed to similar environmental factors, we performed convergent evolution analyses. Convergent sites include both “parallel” and“convergent” sites as defined by J. Zhang and S. Kumar [47]. We reestimated the branch length and reconstructed the ancestral amino acid sequences using the Codeml program in PAML packages [44]. We identified convergent and parallel amino acid substitutions between each pair of species with the following criteria: (1) amino acid residues of both extant species in one pair were identical; (2) amino acid change occurred between the extant lineages and their most recent common ancestor. We then performed a Poisson test to verify whether the observed number of convergent sites of each gene was significantly more than the expected number caused by random substitution under the gcpREV-f_gene_ amino acid substitution models [48, 49]. It has been reported that gcpREV-f_gene_ model performs well using small number of sequences for convergent analyses, although it could cause false positive detection of excessive amount of convergence [49]. To alleviate the possible false positive detection, we used a stringent criterion that the positively selected gene with nonrandom convergent amino acid substitutions is a conservative signature of adaptive evolution [50, 51]. We also applied the FDR correction to the *P* values calculated above to account for the multiple tests performed with a significance level of 0.05 [45].

### Structural analyses of photosynthetic genes under adaptive evolution

In order to gain more insights into the influence of nonrandom convergent substitutions and positively selected sites on the structure and function of the chloroplast proteins, we performed structural analyses using available X-ray crystal structure data. The secondary structures of chloroplast genes with conservative signatures of adaptive evolution in the *Chlamydomonas* sp. ICE-L were predicted using Phyre2 server. Phyre2 is an upgrade to the original Protein Homology/analogY Recognition Engine [52]. The three-dimensional (3D) structures were predicted using the homology modeling software provided by the I-TASSER server [53]. The functional information were derived from the Uniprot (http://www.uniprot.org/). We aligned genes with its homologous protein in *Chlamydomonas reinhardtii* and *Arabidopsis thaliana*. The nonrandom convergent substitutions and positively selected sites were mapped on the predicted secondary and 3D structures to visualize the functional locations using ePlant Web server [54].

## Results

### Positive selection on photosynthetic chloroplast genes

**Fig. 1:**
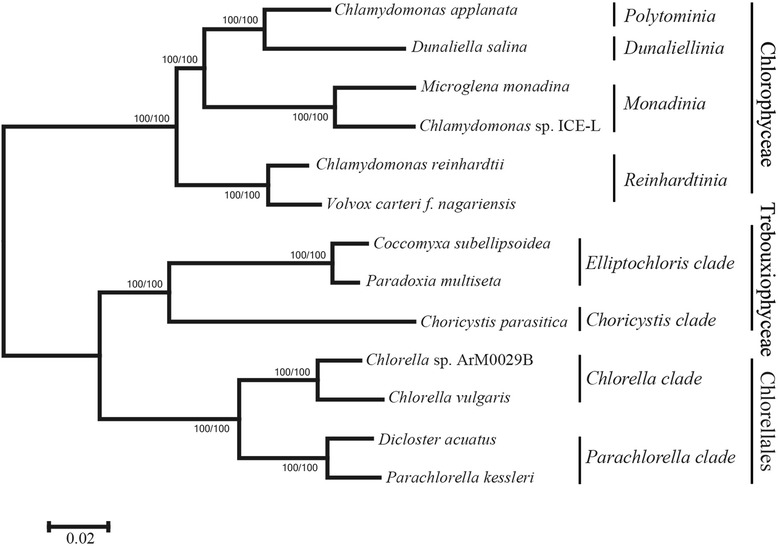
Phylogenetic tree of green algae based on nucleotide data (first and second codon positions) and amino acid data using maximum likelihood method. The bootstrap support values for both data sets are shown on the nodes from left to right

The maximum-likelihood trees confirm that Chlamydomonadales (Chlorophyceae) comprise multiple clades [55, 56], and *Chlamydomonas* sp. ICE-L is a sister group to *Microglena monadina* with high bootstrap support (100%) based on 1st + 2nd codon positions and amino acid data (Fig. 1). The divergence time analyses demonstrate that *Chlamydomonas* sp. ICE-L originated in Permian period (240.7Mya; 95% confidence interval: 138.7~ 379.9Mya) (Additional file 1: Figure S1).

In order to test whether the evolutionary patterns of chloroplast protein-coding genes in *Chlamydomonas* sp. ICE-L are related to extreme environmental adaptation, four algae living in extreme environments were selected for comparative evolutionary analyses. We first tested the basic model (invariant ω across both sites and branches), and found that gene-specific estimates of ω range from 0.00096 to 0.057. All 46 gene-specific ω values were less than 1 (Additional file 1: Table S1). The average of all 46 gene-specific ω values was 0.02999. Only 9 out of 28 photosynthetic genes and 12 out of 18 genetic system genes have larger ω values than the average, respectively.

The branch model test was used to detect positive selection using gene-specific data in *Chlamydomonas* sp. ICE-L. There was no evidence of positive selection in branch model analyses (corrected *P*-value =1 and ω < 1; Additional file 1: Table S2), indicating purifying selection is dominant in chloroplast protein-coding genes. Similarly, there was no evidence of positive selection considering the other three green algae (*Coccomyxa subellipsoidea* C-169, *Chlorella* sp. ArM0029B and *Dunaliella salina*) living in extreme environments as foreground (Additional file 1: Table S2). When using two concatenated data based on two functional classifications (photosynthesis and genetic system), in no case did the test reveal evidence for positive selection in any of the four algae (*Chlamydomonas* sp. ICE-L, *Coccomyxa subellipsoidea* C-169, *Chlorella* sp. ArM0029B and *Dunaliella salina*) (Additional file 1: Table S2).

The random-site model, which ignores ω variation among lineages, was used to identify whether some sites were the targets of positive selection. To test site-specific positive selection in each gene, we used a pair of site models (M8a vs. M8) and FDR correction to the multiple LRT comparisons (corrected *P*-value < 0.05). There was no specific site identified under positive selection using the Bayes empirical Bayes (BEB) method, confirming that purifying selection is predominant force shaping the evolution of chloroplast genes of green algae (Additional file 1: Table S3). Moreover, the examination of site-specific posterior mean ω across two concatenated data showed that ω values were nearly zero for almost all sites. There are a few of sites with ω value more than 0.5 (Additional file 1: Figure S2) implying possible weak purifying selection or episodic positive selection operating at these sites [42].

Branch-site analyses of gene-specific data identified the evidence of positive selection along the lineages leading to *Chlamydomonas* sp. ICE-L, *Dunaliella salina* and *Chlorella* sp. ArM0029B with posterior probabilities ≥0.95 using a Bayes empirical Bayes (BEB) method (Table 2) [46]. BEB analyses identified positively selected genes: three (*atpB*, *psaB*, and *rbcL*) in *Chlamydomonas* sp. ICE-L, three (*atpA*, *atpB*, and *psbC*) in *Dunaliella salina* and two (*atpE* and *petA*) in *Chlorella* sp. ArM0029B. The LRT of branch-site model in three genes (*atpB*, *psbC* and *psbD*) in *Coccomyxa subellipsoidea* C-169 were statistically significant after correction, but there was no positively selected site identified in BEB analyses (PP > 0.95). The same result was obtained in *atpB* gene in *Chlorella* sp. ArM0029B. A total of 10 and 19 positively selected sites were separately identified in *Chlamydomonas* sp. ICE-L and *Dunaliella salina*, whereas only 3 positively selected sites in *Chlorella* sp. ArM0029B. More importantly, all positively selected genes were photosynthetic and encoded vital photosynthetic proteins which are sensitive to abiotic stresses.

**Table 2:** Positively selected sites in corresponding genes of *Chlamydomonas* sp. ICE-L, *Dunaliella salina* and *Chlorella* sp. ArM0029B

Gene	Branch-site model	-lnL	2δ(lnL)	Q Value	ω Values	Positively selected sites
*atpA*	Branch (*Dunaliella salina*)					
	Null	9513.725			ω0 = 0.026, ω1 = 1, ω2 = 1	
	Alternative	9503.900	19.650	4.28E-04	ω0 = 0.026, ω1 = 1, ω2 = 49.199	259–0.994, 269–0.951, 373–0.977, 382–0.996, 426–0.969, 429–0.978, 432–0.992, 434–0.998, 450–0.952, 455–0.978
*atpB*	Branch (*Chlamydomonas* sp. ICE-L)					
	Null	8590.140			ω0 = 0.023, ω1 = 1, ω2 = 1	
	Alternative	8567.141	45.998	5.43E-10	ω0 = 0.022, ω1 = 1, ω2 = 999	24–1.000, 107–0.978, 109–0.999, 375–0.998,
	Branch (*Dunaliella salina*)					
	Null	8566.141			ω0 = 0.021, ω1 = 1, ω2 = 1	
	Alternative	8558.602	15.079	2.37E-03	ω0 = 0.021, ω1 = 1, ω2 = 57.824	112–0.975, 297–0.970, 305–0.970, 331–0.966, 371–0.977
*atpE*	Branch (*Chlorella* sp. ArM0029B)					
	Null	2572.548			ω0 = 0.030, ω1 = 1, ω2 = 1	
	Alternative	2568.079	8.936	4.29E-02	ω0 = 0.030, ω1 = 1, ω2 = 694.685	100–0.987
*petA*	Branch (*Chlorella* sp. ArM0029B)					
	Null	5724.705			ω0 = 0.042, ω1 = 1, ω2 = 1	
	Alternative	5720.106	9.198	4.29E-02	ω0 = 0.042, ω1 = 1, ω2 = 999	216–0.984, 243–0.986
*psaB*	Branch (*Chlamydomonas* sp. ICE-L)					
	Null	12,670.404			ω0 = 0.015, ω1 = 1, ω2 = 1	
	Alternative	12,665.032	10.746	1.60E-02	ω0 = 0.016, ω1 = 1, ω2 = 998.999	220–0.972, 356–0.985, 646–0.990
*psbC*	Branch (*Dunaliella salina*)					
	Null	7158.342			ω0 = 0.019, ω1 = 1, ω2 = 1	
	Alternative	7151.779	13.126	4.47E-03	ω0 = 0.019, ω1 = 1, ω2 = 999	207–0.958, 355–0.988, 360–0.980, 410–0.992
*rbcL*	Branch (*Chlamydomonas* sp. ICE-L)					
	Null	6852.947			ω0 = 0.016, ω1 = 1, ω2 = 1	
	Alternative	6843.536	18.822	3.31E-04	ω0 = 0.017, ω1 = 1, ω2 = 365.261	372–0.969, 450–0.999, 457–0.994

The number for amino acid residues identified by Bayes empirical bayes (BEB) analyses corresponds to their alignment positions. Number behind hyphen is the posterior probability (PP) under BEB analysis

The branch-site analyses of two concatenated data identified signatures of positive selection in seven genes (*atpB*, *psaA*, *psaB*, *psbA*, *psbD*, *rbcL* and *rpl2*; 10 sites in total) in *Chlamydomonas* sp. ICE-L, 12 genes (*atpA*, *atpB*, *petA*, *psaA*, *psbC*, *psbD*, *ccsA*, *chlN*, *rpl5*, *rpl23*, *rps4* and *ycf12*; 30 sites in total) in *Dunaliella salina* (Additional file 1: Table S4). Although the amounts of positively selected genes based on concatenated data are different from that of gene-specific data, it consistently showed that most positively selected sites (9/10 and 23/30) were within photosynthetic genes in *Chlamydomonas* sp. ICE-L and *Dunaliella salina*, respectively. There was no evidence of positive selection for *Coccomyxa subellipsoidea* C-169 and *Chlorella* sp. ArM0029B. It is presumable that these discrepancies based on gene-specific and concatenated data reflect a trade-off between the benefit of increased data and the cost of shared parameter estimates across genes in the large concatenated data compared to the small gene-specific data. It is undeniable that the concatenated data analyses confirmed the results from gene-specific analyses.

In summary, positive selection analyses using both gene-specific and concatenated data revealed putatively adaptive evolutionary patterns of chloroplast protein-coding genes in green algae living in extreme environments: they show in a functional-specific and lineage-specific manner that positive selection mainly target the photosynthetic genes in *Chlamydomonas* sp. ICE-L and *Dunaliella salina.* These genes under positive selection were used in subsequent convergent analyses to discover conservative signatures of adaptive evolution.

### Convergent evolution of *Chlamydomonas* sp. ICE-L in high salinity condition

Adaptive convergence means two or more distant lineages evolve similar traits independently due to similar environmental factors [57]. We identified nonrandom convergent and parallel amino acid substitutions between two halotolerant algae (*Chlamydomonas* sp. ICE-L and *Dunaliella salina*). A total of 19 parallel substitutions from five genes (*petA*, *psbF*, *psbZ*, *rbcL*, *tufA*) and four convergent substitutions from four genes (*atpB*, *atpI*, *petA*, *psaB*) showed significant convergence between *Chlamydomonas* sp. ICE-L and *Dunaliella salina* (Additional file 1: Table S5). Seven convergence genes were photosynthetic, and one gene (*tufA*) encoding elongation factor Tu was responsible for catalyzing the binding of an aminoacyl-tRNA (aa-tRNA) to the ribosome. Consistent with the result of positive selection, it showed same functional specificity that convergent evolution mainly targets photosynthetic genes. To unravel more conservative signatures of adaptive evolution, we focused on positively selected genes with nonrandom convergent amino acid substitutions. As a result, three genes (*atpB*, *psaB*, and *rbcL*) with both signatures of positive selection and convergent evolution were identified although positively selected sites were not overlapped with those that undergo convergent evolution (Table 3). Considering the similar high-salinity environments of *Chlamydomonas* sp. ICE-L and *Dunaliella salina*, we speculate that the conservative signature of adaptive evolution on these genes is associated with acclimation of salt stress.

**Table 3:** Convergent amino acid substitutions between *Chlamydomonas* sp. ICE-L and *Dunaliella salina*

Gene	Function	*P* value^a^	Q-value	Position(s)	AA Changes
*atpB*	ATP synthase subunit beta, chloroplastic	3.18E-03	0.0365	2	Val-Ile
*psaB*	Photosystem I P700 chlorophyll a apoprotein A2	1.27E-04	0.0058	116	Ser-Ala
*rbcL*	Ribulose bisphosphate carboxylase large chain	1.95E-04	0.0019	32	Arg-Ser
				116	Met-Leu
				141	Pro-Ser

The numbering of amino acid residues identified by convergent evolution analyses corresponds to their alignment positions. ^a^: A statistical test is conducted under the assumption that the number of convergent (or parallel) sites follows a Poisson distribution with the mean equal to the expected number. When the observed number is smaller than the expected, the lower tail probability is given; when the observed number is larger than the expected, the upper tail probability is given

### Structural and functional analyses

**Fig. 2:**
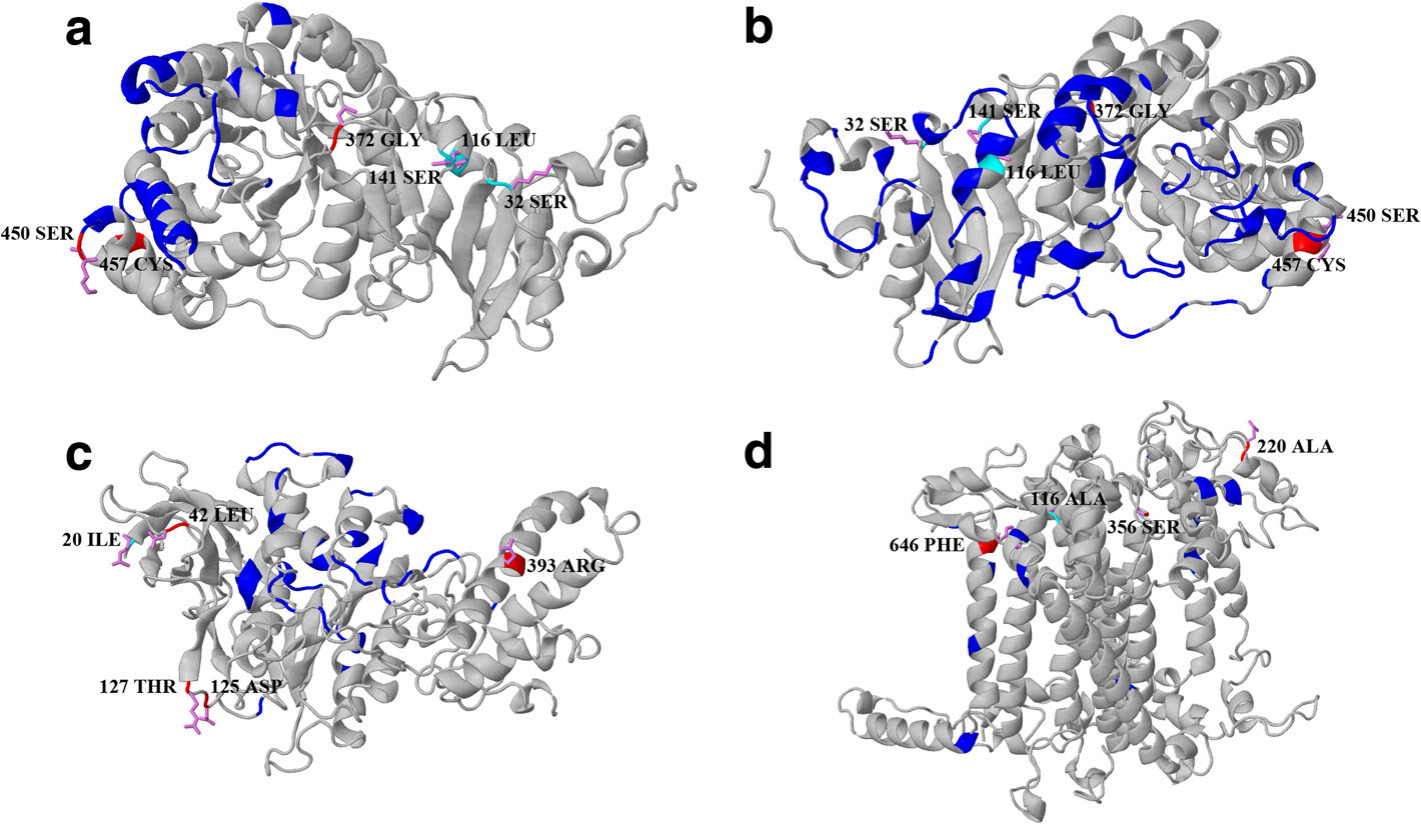
Spatial distribution of positively selected sites and nonrandom convergent substitutions identified on *Chlamydomonas* sp. ICE-L. Positively selected sites (red) and nonrandom convergent substitutions (cyan) were close to functional domains in *Chlamydomonas* sp. ICE-L. The labels of positively selected sites and nonrandom convergent substitutions correspond to the positions in homologous proteins of *Arabidopsis thaliana*. **a** Nonrandom convergent substitutions in *rbcL* gene near the heterodimer interface (in blue). **b** Positively selected sites in *rbcL* gene close to the homodimer interface (in blue). **c** Positively selected site in *atpB* gene near the alpha subunit interaction interface (in blue). **d** Positively selected sites in *psaB* gene close to the predicted carbohydrate binding sites (in blue)

Both positively selected sites and nonrandom convergent substitutions were mapped onto the three-dimensional (3D) crystal structure. Most identified positively selected sites and nonrandom convergent substitutions in *Chlamydomonas* sp. ICE-L were localized in or close to critical residues and functional regions, indicating that many sites were functionally relevant. In *rbcL* gene, the positively selected sites SER_450_ and CYS_457_ were close to the heterodimer interface (TRP_451_ and PRO_453_) at the carboxy-terminus of the large subunit, and nonrandom convergent amino acid substitutions LEU_116_ and SER_141_ were one and nine amino acids from the homodimer interface (ASN_115_ and ALA_132_) (Fig. 2a, b). The positively selected site ASP_125_ in *atpB* gene was five amino acids from the alpha subunit interaction interface position ASN_120_ (Fig. 2c). As for *psaB* gene, the positively selected site PHE_646_ localized in the transmembrane domain (position 644–666 amino acid), and it is eight amino acids from cofactors Mg^2+^ binding site HIS_655_ (position 655 amino acid). In addition, the positively selected site ALA_220_ of *psaB* gene were 10 amino acids from the predicted carbohydrate binding sites ASN_210_ (Fig. 2d).

## Discussion

Photosynthesis is a vital energy accumulation process for algae, and different abiotic stresses may drive adaptive modifications of photosynthetic proteins. The chloroplast protein-coding genes of algae living in extreme environments were used to conduct comparative analyses, aiming to uncover their relationship to the adaptability of extreme environments.

The branch model tests of both gene-specific and concatenated data showed no significant evidence of positive selection targeting the chloroplast protein-coding genes, which indicated strong purifying selection (ω < 1). In general, the functional genes are highly conservative and positive selection may only target specific sites on specific branches, thus positive selection is rare in the branch model test. The random-site model tests based on gene-specific and concatenated data showed that no positively selected site was identified by BEB analyses, implying that purifying selection is the predominant force shaping the evolution of algae chloroplast genes. Two possible reasons were deduced. One reason is that the chloroplast protein-coding genes are conserved to maintain photosynthetic function, and another reason is that the positive selection may have operated on some sites in specific lineages intermittently.

As expected, the notion that positive selection may target specific sites in specific lineages was confirmed through the branch-site model test. In our analyses, only 3 sites were identified under positive selection on *Chlorella* sp. ArM0029B and no site on *Coccomyxa subellipsoidea* C-169, whereas a large number of sites were identified as such along *Chlamydomonas* sp. ICE-L and *Dunaliella salina* in gene-specific and concatenated data analyses. Our results showed that positive selection exclusively targeted chloroplast photosynthetic protein-coding genes along *Chlamydomonas* sp. ICE-L and *Dunaliella salina* lineages.

Considering the observed similar physiological characteristics (salt-tolerant) in *Chlamydomonas* sp. ICE-L and *Dunaliella salina*, elevated salinity is likely the motivation of the adaptive molecular evolution of chloroplast protein-coding genes. Our convergent analyses demonstrated that seven out of eight convergent genes between *Chlamydomonas* sp. ICE-L and *Dunaliella salina* were photosynthetic showing their functional specificity. Why do different evolutionary patterns appear in four algae adapted to extreme environments? One possibility is that adaptive evolution of photosynthetic system may involve in nuclear-encoded subunits. We note that the chloroplast genome is not functionally independent of the nuclear genome [9]. Antenna complexes (nuclear-encoded) experienced adaptive modifications reducing light absorption efficiency and further decreasing the probability of damage to photosystem [58]. Another possible explanation is that adaptive modifications on other abiotic stresses targeting genes were sufficient to maintain the homeostasis for photosynthesis without the need of adaptive evolution of chloroplast-encoded photosynthetic subunits [59, 60].

**Fig. 3:**
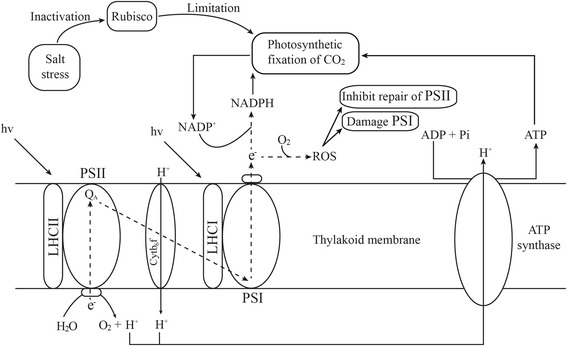
A hypothetical scheme for limitation of photosynthesis under salt stress. When the Rubisco is inactivated by high salt stress, the photosynthetic fixation of CO_2_ is limited. The depletion of NADP^+^ leads to electrons transferring to O_2_. The excess ROS (reactive oxygen species) can damage the PSI and inhibit the repair of PSII

The three-dimensional (3D) crystal structure analyses revealed more insights into how the adaptive sites may influence the function and structure of the chloroplast photosynthetic proteins. Here we focus on the positively selected genes (*rbcL*, *psaB* and *atpB*) with convergent amino acid substitutions in *Chlamydomonas* sp. ICE-L. The *rbcL* gene encoding Rubisco was identified with conservative signatures of adaptive evolution, and numerous adaptive modifications detected in our analyses may be responsible for high salinity stress. The Rubisco is one of the essential enzymes for inorganic carbon fixation in photosynthesis and the primary target of salt stress. High concentration of NaCl may limit the activity of Rubisco, which would inhibit the fixation of CO_2_ (Fig. 3) [15, 61, 62]. Limitation of the photosynthetic fixation of CO_2_ can induce the production of ROS [63]. ROS is mainly generated in plant chloroplast, which could be enhanced under salt stress. It is reported that ROS produced under salt stress decreases photosynthetic rate and carboxylation efficiency resulting in the limitation of photosynthesis [64, 65]. The nonrandom convergent substitutions (SER_32_, LEU_116_ and SER_141_) and positively selected sites (SER_450_ and CYS_457_) in *rbcL* gene were near the homodimer/heterodimer interface, which might contribute to the stability and activity of the enzyme [66]. It is likely that the Rubisco in *Chlamydomonas* sp. ICE-L evolved to restore activity and adapt to abiotic stresses (e.g. high salinity).

Moreover, the ROS is produced at the PSI reaction center, which in turn damage the PSI [67]. Protecting PSI from photodamage in excess ROS generated under salt stress is essential [68]. It is reasonable to assume that *Chlamydomonas* sp. ICE-L have some adaptive modifications to increase resistance to photodamage. For *psaB* gene, adaptive positively selected site PHE_647_ is close to the cofactors Mg^2+^ binding site HIS_655_ possibly contributing to maintain the PSI intactness for normal energy transfer efficiency. The PSI reaction center polypeptides PsaA and PsaB bind many electron transport components [69–71]. Our results showed that *psaB* genes involved in phototrophic electron transport chain experienced adaptive evolution which may help to maintain the stabilization of PSI in *Chlamydomonas* sp. ICE-L.

We also found that *atpB* gene associated with ATP syntheses in chloroplast were under adaptive evolution (Table 3). The beta subunits of ATP synthase encoded by *atpB* gene is responsible for catalysis of ATP synthesis. The positively selected site ASP_125_ in *atpB* gene was near the alpha/beta subunits interaction interface ASN_120_. According to available functional information, this adaptive modification may contribute to the activity of ATP synthase, and the increased ATP synthase activity in *Chlamydomonas* sp. ICE-L could meet the need of CO_2_ fixation. More structural and functional information are essential to gain more insights into the adaptive modifications in *atpB* gene. Together, these confirmative molecular evidences demonstrated that *Chlamydomonas* sp. ICE-L might evolve to maintain high photosynthetic rate and carboxylation efficiency under high salinity environment, and the adaptive modifications in photosynthetic genes of *Chlamydomonas* sp. ICE-L respond to selection pressure from salt stress.

It is noted that both positive selection and convergence are detected at different sites in genes that have undergone adaptive evolution. There are multiple adaptive strategies that influence the adaptive substitutions, and it is reasonable that putatively adaptive substitutions in different green algae living in extreme environments is not convergent in the current study. Similarly, a recent work involving convergent evolution of marine mammals revealed no significant positive correlation between convergence and positive selection in marine mammals [72]. In addition, there are different types of convergent evolution, such as sequence, functional, mechanistic and structural convergence [73]. A previous study has shown strong pattern of convergence emerged at the level of genes, operons, and functional complexes in *Escherichia coli* [74]. We believe that further functional and structural analyses of these adaptive substitutions in *Chlamydomonas* sp. ICE-L will complement our findings.

## Conclusions

We investigate adaptive mechanisms of chloroplast photosynthetic genes from the Antarctic green alga *Chlamydomonas* sp. ICE-L. Our study provides strong evidences that positive Darwinian selection operates on the chloroplast protein-coding genes of *Chlamydomonas* sp. ICE-L adapted to extreme environment following functional-specific and lineages-specific patterns. We identified three genes with conservative signatures of adaptive evolution in *Chlamydomonas* sp. ICE-L, and the adaptive modifications in these genes were in functionally important regions of the proteins. Our results reveal the phototrophic component of *Chlamydomonas* sp. ICE-L exhibits adaptive evolution under extreme environments, and high salinity is likely an important trigger of the adaptations at the chloroplast level. It should be noted that some subunits of photosynthetic systems are nuclear genome-encoded, and it would be more beneficial to understand the adaptive strategies by analyzing all photosynthetic genes in both chloroplast and nuclear genomes. Further experimental and structural analyses will provide confirmative evidences uncovering the adaptive evolution of photosynthetic systems in *Chlamydomonas* sp. ICE-L.

## Additional file

Additional file 1: **Table S1.** Results of the basic (M0) model test of individual genes and the concatenated data. **Table S2.** Results of the correct *P*-value of LRT in branch-model test. **Table S3.** Results of site-model test to each gene-specific and the concatenated data. **Table S4.** Results of branch-site model analyses using the concatenated data. **Table S5.** Results of convergence test between *Chlamydomonas* sp. ICE-L and *Dunaliella salina*. **Figure S1.** Results of divergence time analyses. The estimated divergence times were showed with 95% confidence intervals. **Figure S2.** The dN/dS estimates for the concatenated data using the M8 random-site model. The y-axis shows the BEB posterior mean estimate of dN/dS for each site. (DOCX 341 kb)

## Abbreviations

BEB: Bayes empirical Bayes
BS: bootstrap support
LHCs: Light-harvesting complexes
LRT: likelihood ratio test
Phyre: Protein Homology/analogY Recognition Engine
PP: posterior probability
ROS: Reactive oxygen species
Rubisco: Ribulose bisphosphate carboxylase/oxigenase
