# Supplementary material for: The Antarctic sea ice alga Chlamydomonas sp. ICE-L provides insights into adaptive patterns of chloroplast evolution
Source: BMC Plant Biol. 2018 Apr 3;18:53. doi: 10.1186/s12870-018-1273-x (PMC5883279; doi:10.1186/s12870-018-1273-x)
Supplement: Supplementary file 1 — Table S1. Results of the basic (M0) model test of individual genes and the concatenated data. Table S2. Results of the correct P-value of LRT in branch-model test. Table S3. Results of site-model test to each gene-specific and the concatenated data. Table S4. Results of branch-site model analyses using the concatenated data. Table S5. Results of convergence test between Chlamydomonas sp. ICE-L and Dunaliella salina. Figure S1. Results of divergence time analyses. The estimated divergence times were showed with 95% confidence intervals. Figure S2. The dN/dS estimates for the concatenated data using the M8 random-site model. The y-axis shows the BEB posterior mean estimate of dN/dS for each site. (DOCX 341 kb) [file 12870_2018_1273_MOESM1_ESM.docx]

**Supplementary Materials and Methods**

*Divergence time estimation*

The MCMCTREE in PAML v4.9 [[1](#_ENREF_1)] was used to estimate divergence times with one fossil calibration point applied: the fossil-based divergence time (split time) of Chlorophyceae and Ulvophyceae (min = 750 Mya, max =1200 Mya) [[2](#_ENREF_2)]. The likelihood was calculated under the GTR + Γ4 model using the method of normal approximation [[3](#_ENREF_3)], and the independent rates (IR) model was used. The prior on rate was set to G (1,10), and the prior for σ^2^ was set to G(1, 12). The MCMC was run for 2*10^4^ iterations, sampling every 1000 iterations. The first 1*10^6^ iterations was discarded as burn-in. Two independent runs were launched to ensure convergence.

Reference:

1. Yang Z: **PAML 4: Phylogenetic Analysis by Maximum Likelihood**. *Molecular biology and evolution* 2007, **24**(8):1586-1591.

2. Teyssedre B: **Precambrian palaeontology in the light of molecular phylogeny–an example: the radiation of the green algae**. *Biogeosciences Discussions* 2007, **4**(5):3123-3142.

3. Reis Md, Yang Z: **Approximate likelihood calculation on a phylogeny for Bayesian estimation of divergence times**. *Molecular biology and evolution* 2011, **28**(7):2161-2172.

**Supplementary Tables**

**Table S1.** Results of the basic model (model=0, NSsites=0) test of individual genes and concatenated data.

| **Function** | **Gene** | **ω** | **lnL** | **Branch-specific dS estimates** | | | **Alignment Length(bp)** |
| --- | --- | --- | --- | --- | --- | --- | --- |
|  |  |  |  | **Min** | **Max** | **Median** |  |
| Photosynthetic | *atpA* | 0.04 | -9650.344 | 0.091 | 1.611 | 0.366 | 1503 |
| genes | *atpB* | 0.03 | -8682.938 | 0.074 | 1.549 | 0.380 | 1413 |
|  | *atpE* | 0.04 | -2594.888 | 0.016 | 3.246 | 0.473 | 387 |
|  | *atpF* | 0.06 | -3822.675 | 0.056 | 3.698 | 0.500 | 477 |
|  | *atpH* | 0.00 | -646.197 | 0.000 | 2.043 | 0.216 | 165 |
|  | *atpI* | 0.03 | -4576.467 | 0.000 | 1.748 | 0.619 | 690 |
|  | *petA* | 0.05 | -5740.968 | 0.211 | 1.336 | 0.456 | 834 |
|  | *petB* | 0.02 | -3382.082 | 0.000 | 2.215 | 0.335 | 642 |
|  | *petD* | 0.02 | -2787.372 | 0.000 | 1.376 | 0.460 | 477 |
|  | *petG* | 0.01 | -599.103 | 0.000 | 4.068 | 0.480 | 105 |
|  | ***petL*** | **0.06** | **-657.869** | **0.000** | **5.503** | **0.453** | **93** |
|  | *psaA* | 0.02 | -12225.400 | 0.067 | 1.371 | 0.352 | 2250 |
|  | *psaB* | 0.02 | -12825.898 | 0.020 | 1.597 | 0.444 | 2196 |
|  | ***psaJ*** | **0.00** | **-768.993** | **0.000** | **84.410** | **6.757** | **120** |
|  | *psbA* | 0.03 | -4776.977 | 0.000 | 0.415 | 0.164 | 1053 |
|  | *psbB* | 0.03 | -8381.221 | 0.050 | 1.351 | 0.340 | 1515 |
|  | *psbC* | 0.02 | -7189.194 | 0.086 | 0.974 | 0.345 | 1362 |
|  | *psbD* | 0.02 | -5149.972 | 0.020 | 1.101 | 0.314 | 1056 |
|  | *psbE* | 0.02 | -1135.194 | 0.000 | 1.330 | 0.246 | 240 |
|  | *psbF* | 0.02 | -481.705 | 0.000 | 1.627 | 0.201 | 102 |
|  | ***psbH*** | **0.02** | **-1311.821** | **0.000** | **6.900** | **0.703** | **198** |
|  | *psbI* | 0.01 | -496.702 | 0.000 | 4.606 | 0.424 | 99 |
|  | *psbJ* | 0.06 | -791.413 | 0.000 | 4.610 | 0.135 | 120 |
|  | *psbK* | 0.02 | -572.603 | 0.000 | 2.263 | 0.251 | 111 |
|  | *psbL* | 0.01 | -495.262 | 0.000 | 2.881 | 0.200 | 111 |
|  | *psbM* | 0.02 | -484.499 | 0.000 | 4.387 | 0.220 | 96 |
|  | *psbN* | 0.01 | -648.634 | 0.000 | 4.602 | 0.375 | 132 |
|  | *psbT* | 0.03 | -471.631 | 0.000 | 0.984 | 0.277 | 93 |
|  | *psbZ* | 0.04 | -1114.246 | 0.000 | 2.838 | 0.283 | 183 |
|  | *rbcL* | 0.03 | -6972.007 | 0.021 | 0.785 | 0.229 | 1422 |
|  | *ycf12* | 0.06 | -693.944 | 0.000 | 2.865 | 0.344 | 96 |
|  | ***ycf3*** | **0.00** | **-2715.150** | **0.000** | **8.100** | **0.919** | **495** |
|  | ***ycf4*** | **0.04** | **-4142.101** | **0.240** | **6.755** | **0.669** | **504** |
|  | pho_con | 0.03 | -108654.805 | 0.078 | 1.140 | 0.318 | 18834 |
| Genetic | *ccsA* | 0.03 | -5075.839 | 0.017 | 4.546 | 1.112 | 645 |
| system genes | ***cemA*** | **0.01** | **-4051.841** | **0.000** | **21.904** | **1.354** | **543** |
|  | *chlB* | 0.02 | -9138.858 | 0.063 | 3.168 | 0.817 | 1377 |
|  | ***chlL*** | **0.01** | **-4556.782** | **0.000** | **5.204** | **1.046** | **780** |
|  | *chlN* | 0.02 | -7528.060 | 0.080 | 2.633 | 0.740 | 1158 |
|  | ***clpP*** | **0.01** | **-3146.040** | **0.000** | **47.454** | **0.712** | **468** |
|  | *rpl14* | 0.02 | -2012.657 | 0.000 | 4.571 | 0.477 | 822 |
|  | *rpl16* | 0.04 | -2674.859 | 0.096 | 2.067 | 0.425 | 531 |
|  | *rpl20* | 0.05 | -2589.887 | 0.000 | 2.447 | 0.588 | 339 |
|  | *rpl23* | 0.04 | -1903.965 | 0.047 | 3.158 | 0.777 | 393 |
|  | *rpl2* | 0.04 | -6352.233 | 0.252 | 2.249 | 0.519 | 324 |
|  | *rpl36* | 0.02 | -593.792 | 0.000 | 3.756 | 0.704 | 225 |
|  | *rpl5* | 0.04 | -3726.237 | 0.060 | 2.859 | 0.607 | 108 |
|  | ***rps11*** | **0.03** | **-2915.504** | **0.086** | **5.242** | **0.502** | **102** |
|  | *rps12* | 0.02 | -2280.678 | 0.102 | 2.295 | 0.544 | 408 |
|  | *rps14* | 0.05 | -2422.517 | 0.031 | 3.367 | 0.618 | 444 |
|  | *rps18* | 0.05 | -1384.688 | 0.000 | 3.694 | 0.523 | 354 |
|  | *rps19* | 0.04 | -1752.707 | 0.000 | 2.178 | 0.532 | 309 |
|  | ***rps2*** | **0.02** | **-914.950** | **0.000** | **30.533** | **1.065** | **387** |
|  | *rps4* | 0.04 | -3049.301 | 0.099 | 2.712 | 0.634 | 360 |
|  | ***rps7*** | **0.04** | **-3720.253** | **0.000** | **6.998** | **0.656** | **300** |
|  | *rps8* | 0.04 | -2695.080 | 0.222 | 3.066 | 0.676 | 177 |
|  | *rps9* | 0.05 | -2529.130 | 0.143 | 3.596 | 0.533 | 270 |
|  | *tufA* | 0.03 | -7392.151 | 0.040 | 1.660 | 0.462 | 1212 |
|  | gen_con | 0.03 | -65569.439 | 0.183 | 2.166 | 0.645 | 9255 |

dS: the number of synonymous substitutions per synonymous site; “pho_con”: the concatenated data of photosynthetic genes; “gen_con”: the concatenated data of genetic system genes. The 11 genes (in bold) with high dS value (dS > 5) were discarded to avoid misestimation of dN/dS.

**Table S2.** Results of the correct *P*-value of LRT in branch-model test

| **Gene** | **Initial ω=0.5** | | | | **Initial ω=1** | | | | **Initial ω=2** | | | |
| --- | --- | --- | --- | --- | --- | --- | --- | --- | --- | --- | --- | --- |
|  | **q_ice** | **q_dun** | **q_coc** | **q_chl** | **q_ice** | **q_dun** | **q_coc** | **q_chl** | **q_ice** | **q_dun** | **q_coc** | **q_chl** |
| *atpA* | 0.2655 | 0.0000 | 0.1555 | 0.1892 | 0.2655 | 0.0000 | 0.1668 | 0.1514 | 0.2655 | 0.0000 | 0.1444 | 0.1261 |
| *atpB* | 0.2303 | 0.1420 | 0.0013 | 0.4391 | 0.2303 | 0.1278 | 0.0009 | 0.3903 | 0.2303 | 0.1162 | 0.0007 | 0.3513 |
| *atpE* | 0.4514 | 0.2237 | 0.2387 | 0.8729 | 0.4309 | 0.2065 | 0.2668 | 0.8244 | 0.4309 | 0.1917 | 0.2668 | 0.8235 |
| *atpF* | 0.0629 | 0.4766 | 0.5566 | 0.7056 | 0.0629 | 0.4590 | 0.5752 | 0.6552 | 0.0629 | 0.4426 | 0.5392 | 0.5733 |
| *atpH* | 0.9970 | 0.8592 | 0.9972 | 0.9665 | 0.9972 | 0.0004 | 0.9980 | 0.0385 | 0.9972 | 0.0004 | 0.9972 | 0.0308 |
| *atpI* | 0.8429 | 0.6810 | 0.5057 | 0.7239 | 0.8429 | 0.6597 | 0.5268 | 0.6756 | 0.8639 | 0.6597 | 0.5057 | 0.0308 |
| *ccsA* | 0.9970 | 0.7027 | 0.2324 | 0.7056 | 0.9972 | 0.6826 | 0.2614 | 0.6552 | 0.9972 | 0.6826 | 0.2614 | 0.5733 |
| *chlB* | 0.6985 | 0.7879 | 0.2792 | 0.0021 | 0.6985 | 0.7677 | 0.3086 | 0.0021 | 0.6736 | 0.7677 | 0.2932 | 0.0021 |
| *chlN* | 0.8429 | 0.1420 | 0.2252 | 0.9832 | 0.8429 | 0.1278 | 0.2574 | 0.9363 | 0.9972 | 0.1144 | 0.0008 | 0.9560 |
| *petA* | 0.0326 | 0.8258 | 0.0018 | 0.8972 | 0.0326 | 0.8052 | 0.0015 | 0.8500 | 0.0326 | 0.8052 | 0.0013 | 0.8500 |
| *petB* | 0.5009 | 0.2237 | 0.2324 | 0.8729 | 0.7303 | 0.2065 | 0.2614 | 0.8244 | 0.4800 | 0.1917 | 0.2614 | 0.8134 |
| *petD* | 0.8557 | 0.1012 | 0.3022 | 0.8039 | 0.8557 | 0.0809 | 0.3324 | 0.7570 | 0.8816 | 0.0809 | 0.3022 | 0.7235 |
| *petG* | 0.9970 | 0.4163 | 0.5566 | 0.8252 | 0.9972 | 0.3996 | 0.5752 | 0.7570 | 0.9972 | 0.3843 | 0.5392 | 0.7368 |
| *psaA* | 0.3010 | 0.3265 | 0.2666 | 0.0021 | 0.3010 | 0.3084 | 0.2962 | 0.0021 | 0.3010 | 0.2921 | 0.2932 | 0.0021 |
| *psaB* | 0.0000 | 0.5515 | 0.0006 | 0.7056 | 0.0000 | 0.5331 | 0.0004 | 0.6552 | 0.0000 | 0.5331 | 0.0003 | 0.5733 |
| *psbA* | 0.9970 | 0.1319 | 0.0160 | 0.7056 | 0.9972 | 0.1131 | 0.0160 | 0.6552 | 0.9972 | 0.0989 | 0.0160 | 0.5733 |
| *psbB* | 0.1958 | 0.2288 | 0.0775 | 0.4391 | 0.1958 | 0.2135 | 0.0775 | 0.3903 | 0.1958 | 0.2002 | 0.0705 | 0.3513 |
| *psbC* | 0.4341 | 0.1012 | 0.0016 | 0.8729 | 0.4134 | 0.0809 | 0.0012 | 0.8244 | 0.4134 | 0.0809 | 0.0001 | 0.8134 |
| *psbD* | 0.9970 | 0.1012 | 0.0285 | 0.9912 | 0.9972 | 0.0809 | 0.0004 | 0.9980 | 0.9972 | 0.0809 | 0.0003 | 0.9980 |
| *psbE* | 0.5009 | 0.6982 | 0.5041 | 0.8252 | 0.4878 | 0.6776 | 0.5260 | 0.7570 | 0.4800 | 0.6776 | 0.5041 | 0.7368 |
| *psbF* | 0.9970 | 0.3321 | 0.5889 | 0.8252 | 0.9972 | 0.3146 | 0.5889 | 0.7570 | 0.9972 | 0.2989 | 0.5608 | 0.7368 |
| *psbI* | 0.9970 | 0.3418 | 0.9972 | 0.8729 | 0.9972 | 0.3276 | 0.9980 | 0.7570 | 0.9972 | 0.3145 | 0.9972 | 0.7368 |
| *psbJ* | 0.7619 | 0.9745 | 0.5198 | 0.8039 | 0.7373 | 0.9745 | 0.5406 | 0.7570 | 0.7373 | 0.9745 | 0.5198 | 0.7235 |
| *psbK* | 0.9970 | 0.3418 | 0.9972 | 0.9912 | 0.9972 | 0.3276 | 0.9980 | 0.9980 | 0.9972 | 0.3145 | 0.9972 | 0.9980 |
| *psbL* | 0.1821 | 0.1766 | 0.5889 | 0.9665 | 0.1821 | 0.1606 | 0.5889 | 0.9139 | 0.1821 | 0.1472 | 0.5677 | 0.9560 |
| *psbM* | 0.9970 | 0.3418 | 0.5889 | 0.8972 | 0.9972 | 0.3276 | 0.5889 | 0.8500 | 0.9972 | 0.3145 | 0.5392 | 0.8500 |
| *psbN* | 0.7969 | 0.2645 | 0.1555 | 0.9912 | 0.3893 | 0.2479 | 0.4167 | 0.9980 | 0.3893 | 0.2333 | 0.1444 | 0.9980 |
| *psbT* | 0.2525 | 0.7433 | 0.5413 | 0.8729 | 0.2525 | 0.7226 | 0.5614 | 0.8244 | 0.2525 | 0.7226 | 0.5392 | 0.8134 |
| *psbZ* | 0.2303 | 0.1123 | 0.5889 | 0.8729 | 0.2303 | 0.0935 | 0.5889 | 0.8244 | 0.2303 | 0.0935 | 0.5608 | 0.8235 |
| *rbcL* | 0.4341 | 0.3418 | 0.9972 | 0.8024 | 0.4134 | 0.3276 | 0.9980 | 0.7522 | 0.4134 | 0.3145 | 0.9972 | 0.7080 |
| *rpl14* | 0.9970 | 0.7879 | 0.6384 | 0.9912 | 0.9972 | 0.7677 | 0.6385 | 0.8244 | 0.9972 | 0.7677 | 0.2932 | 0.8235 |
| *rpl16* | 0.0231 | 0.9626 | 0.0128 | 0.8039 | 0.0231 | 0.9626 | 0.0128 | 0.7570 | 0.0231 | 0.9626 | 0.0128 | 0.7235 |
| *rpl20* | 0.6443 | 0.5685 | 0.9555 | 0.4391 | 0.6443 | 0.5502 | 0.9555 | 0.3903 | 0.6204 | 0.5502 | 0.9555 | 0.3513 |
| *rpl23* | 0.3010 | 0.6982 | 0.7021 | 0.8729 | 0.3010 | 0.6776 | 0.7021 | 0.8244 | 0.3010 | 0.6776 | 0.7021 | 0.5733 |
| *rpl2* | 0.0651 | 0.5515 | 0.9185 | 0.8252 | 0.0651 | 0.5331 | 0.9341 | 0.7570 | 0.0651 | 0.0989 | 0.9341 | 0.7368 |
| *rpl36* | 0.5699 | 0.4408 | 0.5889 | 0.9912 | 0.5699 | 0.4238 | 0.5889 | 0.9139 | 0.5471 | 0.4081 | 0.5608 | 0.9155 |
| *rpl5* | 0.0326 | 0.9626 | 0.1098 | 0.8729 | 0.0326 | 0.9626 | 0.1098 | 0.8244 | 0.0326 | 0.9626 | 0.1007 | 0.8134 |
| *rps12* | 0.4042 | 0.9626 | 0.0077 | 0.9870 | 0.3893 | 0.9626 | 0.5889 | 0.9411 | 0.3893 | 0.9626 | 0.5608 | 0.9560 |
| *rps14* | 0.9970 | 0.9485 | 0.1803 | 0.4391 | 0.9972 | 0.9485 | 0.0767 | 0.3903 | 0.9972 | 0.9485 | 0.0690 | 0.3513 |
| *rps18* | 0.7217 | 0.1420 | 0.2026 | 0.8252 | 0.7217 | 0.1278 | 0.2338 | 0.7570 | 0.6977 | 0.1162 | 0.3022 | 0.7368 |
| *rps19* | 0.7217 | 0.5515 | 0.3696 | 0.8252 | 0.7217 | 0.5331 | 0.4048 | 0.7570 | 0.6977 | 0.5331 | 0.3696 | 0.7368 |
| *rps4* | 0.4341 | 0.3418 | 0.7927 | 0.7056 | 0.4134 | 0.3276 | 0.8130 | 0.6552 | 0.4134 | 0.3145 | 0.8130 | 0.5733 |
| *rps8* | 0.1972 | 0.2283 | 0.5219 | 0.8729 | 0.1972 | 0.2120 | 0.5419 | 0.8244 | 0.1972 | 0.1979 | 0.5219 | 0.8235 |
| *rps9* | 0.9970 | 0.7652 | 0.7752 | 0.9832 | 0.9972 | 0.7446 | 0.9341 | 0.9363 | 0.9972 | 0.7446 | 0.9341 | 0.9560 |
| *tufA* | 0.0651 | 0.2974 | 0.0018 | 0.0513 | 0.0651 | 0.2799 | 0.0015 | 0.0385 | 0.0651 | 0.2644 | 0.0263 | 0.0308 |
| *ycf12* | 0.6443 | 0.9626 | 0.5566 | 0.8252 | 0.6443 | 0.9626 | 0.5752 | 0.7570 | 0.6204 | 0.9626 | 0.5392 | 0.7368 |
| pho_con | 0.0000 | 0.0000 | 0.0000 | 0.0000 | 0.0000 | 0.0000 | 0.0000 | 0.0000 | 0.0000 | 0.0000 | 0.0000 | 0.0000 |
| gen_con | 0.0000 | 0.9010 | 0.0000 | 0.0000 | 0.0000 | 0.9010 | 0.0000 | 0.0000 | 0.0000 | 0.9010 | 0.0000 | 0.0000 |

The correct *P*-value is the correction of *P* values for the multiple tests. q_ice: correct *P*-value for *Chlamydomonas* sp. ICE-L; q_dun: correct *P*-value for *Dunaliella salina*; q_coc: correct *P*-value for *Coccomyxa* *subellipsoidea* C-169; q_chl: correct *P*-value for *Chlorella* sp. ArM0029B. The “pho_con” is the concatenated data of photosynthetic genes, and “gen_con” is the concatenated data of genetic system genes.

**Table S3.** Results of site-model test to each gene-specific and the concatenated data

| **Gene** | **Alternative model (lnL)** | **Null model (lnL)** | ***P*-value** | **Q-value** |
| --- | --- | --- | --- | --- |
| *atpA* | -9413.298911 | -9413.298911 | 1 | 1 |
| *atpB* | -8422.316781 | -8427.682751 | 0.00105302 | 0.02421946 |
| *atpE* | -2560.127521 | -2560.127521 | 1 | 1 |
| *atpF* | -3754.11593 | -3754.11593 | 1 | 1 |
| *atpH* | -646.205973 | -646.205973 | 1 | 1 |
| *atpI* | -4449.344724 | -4449.344724 | 1 | 1 |
| *ccsA* | -4882.312868 | -4882.312868 | 1 | 1 |
| *chlB* | -8872.180526 | -8872.180526 | 1 | 1 |
| *chlN* | -7279.884629 | -7297.09864 | 4.42309E-09 | 2.03E-07 |
| *petA* | -5635.763767 | -5635.763767 | 1 | 1 |
| *petB* | -3309.907145 | -3308.661863 | 0.114530675 | 1 |
| *petD* | -2745.348286 | -2745.348286 | 1 | 1 |
| *petG* | -592.431871 | -592.431871 | 1 | 1 |
| *psaA* | -12059.92063 | -12059.92063 | 1 | 1 |
| *psaB* | -12523.44353 | -12523.44353 | 1 | 1 |
| *psbA* | -4704.489479 | -4703.689992 | 0.206048671 | 1 |
| *psbB* | -8268.087606 | -8268.087606 | 1 | 1 |
| *psbC* | -7067.31278 | -7067.310776 | 0.949520635 | 1 |
| *psbD* | -5113.315433 | -5113.315055 | 0.978064566 | 1 |
| *psbE* | -1126.233518 | -1126.233506 | 0.996091196 | 1 |
| *psbF* | -478.249393 | -478.249393 | 1 | 1 |
| *psbI* | -493.483344 | -493.483344 | 1 | 1 |
| *psbJ* | -777.208678 | -777.208668 | 0.996431764 | 1 |
| *psbK* | -566.804373 | -566.804368 | 0.997476872 | 1 |
| *psbL* | -485.040895 | -485.141478 | 0.653781326 | 1 |
| *psbM* | -472.545565 | -472.601802 | 0.737344912 | 1 |
| *psbN* | -645.517065 | -645.517065 | 1 | 1 |
| *psbT* | -457.304178 | -457.304178 | 1 | 1 |
| *psbZ* | -1102.691999 | -1102.691999 | 1 | 1 |
| *rbcL* | -6789.24592 | -6789.245931 | 0.996257603 | 1 |
| *rpl14* | -1985.778995 | -1985.48863 | 0.446026326 | 1 |
| *rpl16* | -2638.767479 | -2638.767479 | 1 | 1 |
| *rpl20* | -2533.941327 | -2533.941327 | 1 | 1 |
| *rpl23* | -1872.902654 | -1872.902654 | 1 | 1 |
| *rpl2* | -6194.329169 | -6194.327413 | 0.952743324 | 1 |
| *rpl36* | -587.006477 | -587.006475 | 0.998404232 | 1 |
| *rpl5* | -3641.217071 | -3641.217055 | 0.995486507 | 1 |
| *rps12* | -2254.07487 | -2253.307714 | 0.215466442 | 1 |
| *rps14* | -2351.134847 | -2351.134599 | 0.982231736 | 1 |
| *rps18* | -1369.823342 | -1369.823342 | 1 | 1 |
| *rps19* | -1728.853653 | -1728.853653 | 1 | 1 |
| *rps4* | -2988.14138 | -2988.140945 | 0.976469199 | 1 |
| *rps8* | -2625.52925 | -2625.52925 | 1 | 1 |
| *rps9* | -2473.178086 | -2473.178076 | 0.996431764 | 1 |
| *tufA* | -7177.931541 | -7177.931541 | 1 | 1 |
| *ycf12* | -688.49556 | -688.495547 | 0.995931589 | 1 |
| pho_con | -106295.5793 | -106295.5793 | 1 |  |
| gen_con | -63851.32905 | -63851.32905 | 1 |  |

The “pho_con” is the concatenated data of photosynthetic genes, and “gen_con” is the concatenated data of genetic system genes. *P*-value is the results of LRT test. Q value is the correction to the *P* values for the multiple tests.

**Table S4.** Results of branch-site model analyses using the concatenated data

| **Data sets** | **Branch** | **Branch-site model** | **-lnL** | **ω Values** | | | ***P* Value** | **Positively selected sites** |
| --- | --- | --- | --- | --- | --- | --- | --- | --- |
|  |  |  |  |  |  |  |  | Gene:site |
| pho_con | *Chlamydomonas* sp. ICE-L | Alternative | -107638.63 | ω0=0.025 | ω1=1 | ω2=119.99 | 9.2E-14 | *atpB*: 24,109 |
|  |  | Null | -107665.01 | ω0=0.025 | ω1=1 | ω2=1 |  | *psaA*: 503 |
|  |  |  |  |  |  |  |  | *psaB*: 646 |
|  |  |  |  |  |  |  |  | *psbA*: 268 |
|  |  |  |  |  |  |  |  | *psbD*: 273 |
|  |  |  |  |  |  |  |  | *rbcL*: 328,450,457 |
| gen_con |  | Alternative | -65116.69 | ω0=0.031 | ω1=1 | ω2=3.07 | 0.02085 | *rpl2*: 72 |
|  |  | Null | -65120.08 | ω0=0.031 | ω1=1 | ω2=1 |  |  |
| pho_con | *Dunaliella salina* | Alternative | -107594.40 | ω0=0.025 | ω1=1 | ω2=48.56 | 1.6E-12 | *atpA*: 259,269,373,382,432,434 |
|  |  | Null | -107619.80 | ω0=0.025 | ω1=1 | ω2=1 |  | *atpB*: 77,112,205,297,305,321,331,371 |
|  |  |  |  |  |  |  |  | *petA*: 9,62 |
|  |  |  |  |  |  |  |  | *psaA*: 64,708 |
|  |  |  |  |  |  |  |  | *psbC*: 355,360,410 |
|  |  |  |  |  |  |  |  | *psbD*: 52 |
|  |  |  |  |  |  |  |  | *ycf12*: 4 |
| gen_con |  | Alternative | -65113.90 | ω0=0.031 | ω1=1 | ω2=166.03 | 3.4E-06 | *ccsA*: 27 |
|  |  | Null | -65124.39 | ω0=0.031 | ω1=1 | ω2=1 |  | *chlN*: 248,360,375 |
|  |  |  |  |  |  |  |  | *rpl5*: 167 |
|  |  |  |  |  |  |  |  | *rpl23*: 37 |
|  |  |  |  |  |  |  |  | *rps4*: 99 |

The “pho_con” is the concatenated data of photosynthetic genes, and “gen_con” is the concatenated data of genetic system genes. *P*-value is the results of LRT test. Sites identified as positively selected by Bayes empirical Bayes (BEB) analysis with posterior probability (PP) >= 0.95 are listed.

**Table S5**. The results of convergence test between *Chlamydomonas* sp. ICE-L and *Dunaliella salina*.

| **Gene** | **Protein** | **Parallel  observed  number** | **Parallel  expected number** | **Convergent  observed number** | **Convergent  expected**  **number** | **Q value  for parallel** | **Q value  for convergent** |
| --- | --- | --- | --- | --- | --- | --- | --- |
|  |  |  |  |  |  |  |  |
| *atpB* | ATP synthase subunit beta, chloroplastic | 1 | 0.4963 | 1 | 0.0819 | 0.311745 | 0.036526 |
| *atpI* | ATP synthase subunit IV, chloroplastic | 0 | 0.3072 | 1 | 0.0496 | 1 | 0.018249 |
| *petA* | Cytochrome *f* | 4 | 0.4671 | 1 | 0.0333 | 0.00186 | 0.012473 |
| *psaB* | Photosystem I P700 chlorophyll a apoprotein A2 | 1 | 0.2507 | 1 | 0.016 | 0.111385 | 0.005826 |
| *psbF* | Cytochrome b*559* subunit beta | 1 | 0.0154 | 0 | 8.00E-04 | 0.00186 | 1 |
| *psbZ* | Photosystem II protein Z | 2 | 0.1096 | 0 | 0.0063 | 0.00186 | 1 |
| *rbcL* | Ribulose bisphosphate carboxylase large chain | 3 | 0.2764 | 0 | 0.0145 | 0.00186 | 1 |
| *tufA* | Elongation factor Tu, chloroplastic | 5 | 0.3822 | 0 | 0.0335 | 0.000144 | 1 |

The observed numbers of convergent and parallel sites and the corresponding expected numbers are provided. Q value is the correction to the *P* values for the multiple tests.

**Supplementary Figure**

**Figure S1**


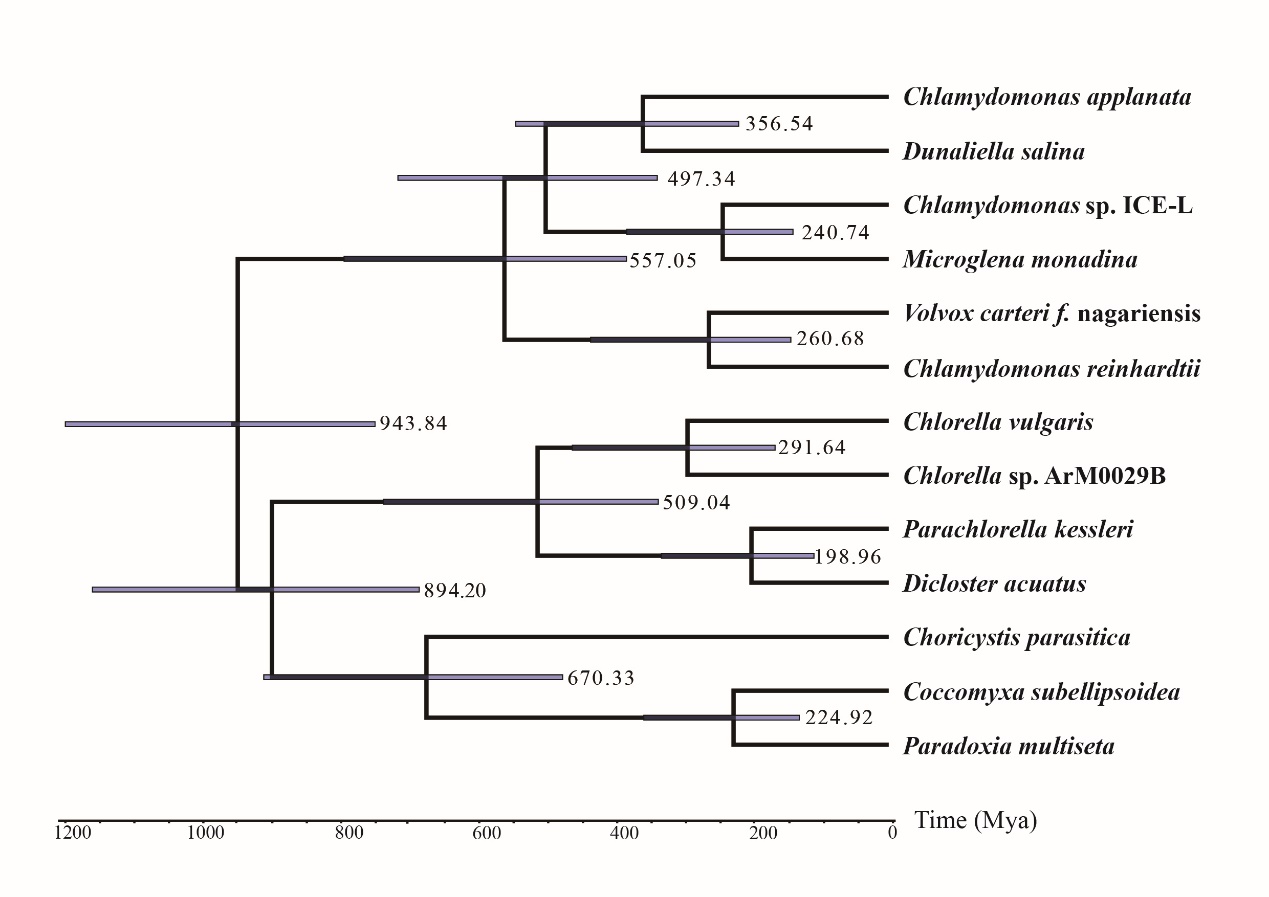


**Figure S2**

**
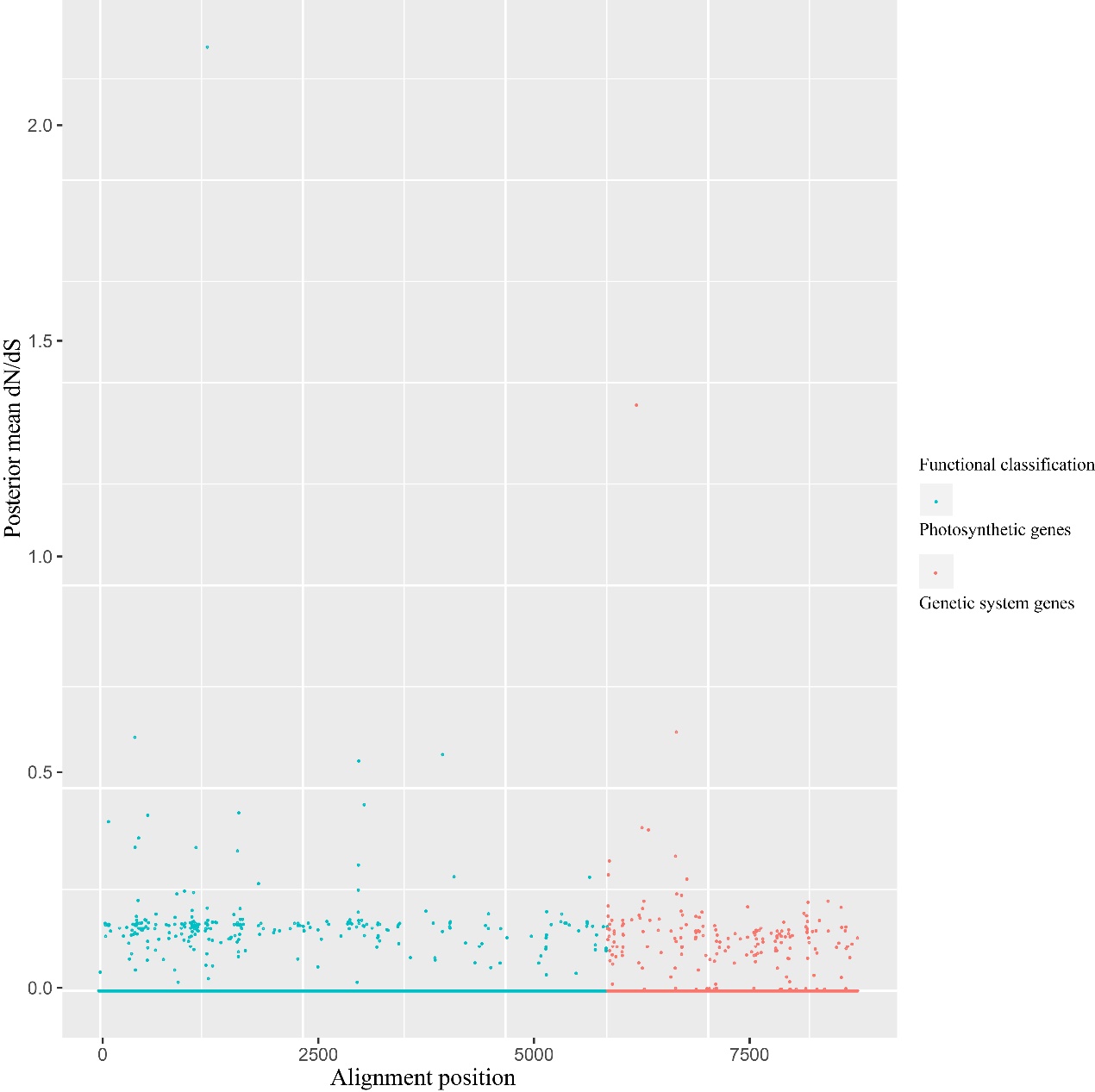
**
